# Lipoprotein Subclasses Independently Contribute to Subclinical Variance of Microvascular and Macrovascular Health

**DOI:** 10.3390/molecules27154760

**Published:** 2022-07-25

**Authors:** Lukas Streese, Hansjörg Habisch, Arne Deiseroth, Justin Carrard, Denis Infanger, Arno Schmidt-Trucksäss, Tobias Madl, Henner Hanssen

**Affiliations:** 1 Department of Sport, Exercise and Health, Medical Faculty, University of Basel, 4052 Basel, Switzerland; lukas.streese@unibas.ch (L.S.); arne.deiseroth@unibas.ch (A.D.); justin.carrard@unibas.ch (J.C.); denis.infanger@unibas.ch (D.I.); arno.schmidt-trucksaess@unibas.ch (A.S.-T.); henner.hanssen@unibas.ch (H.H.); 2 Gottfried Schatz Research Center for Cell Signaling, Metabolism and Aging, Molecular Biology and Biochemistry, Medical University of Graz, 8010 Graz, Austria; hansjoerg.habisch@medunigraz.at; 3 BioTechMed Graz, 8010 Graz, Austria

**Keywords:** cardiovascular risk, lipids, NMR spectroscopy, pulse wave velocity, retinal vessel diameters

## Abstract

Lipoproteins are important cardiovascular (CV) risk biomarkers. This study aimed to investigate the associations of lipoprotein subclasses with micro- and macrovascular biomarkers to better understand how these subclasses relate to atherosclerotic CV diseases. One hundred and fifty-eight serum samples from the EXAMIN AGE study, consisting of healthy individuals and CV risk patients, were analysed with nuclear magnetic resonance (NMR) spectroscopy to quantify lipoprotein subclasses. Microvascular health was quantified by measuring retinal arteriolar and venular diameters. Macrovascular health was quantified by measuring carotid-to-femoral pulse wave velocity (PWV). Nineteen lipoprotein subclasses showed statistically significant associations with retinal vessel diameters and nine with PWV. These lipoprotein subclasses together explained up to 26% of variation (R^2^ = 0.26, F(29,121) = 2.80, *p* < 0.001) in micro- and 12% (R^2^ = 0.12, F(29,124) = 1.70, *p* = 0.025) of variation in macrovascular health. High-density (HDL-C) and low-density lipoprotein cholesterol (LDL-C) as well as triglycerides together explained up to 13% (R^2^ = 0.13, F(3143) = 8.42, *p* < 0.001) of micro- and 8% (R^2^ = 0.08, F(3145) = 5.46, *p* = 0.001) of macrovascular variation. Lipoprotein subclasses seem to reflect micro- and macrovascular end organ damage more precisely as compared to only measuring HDL-C, LDL-C and triglycerides. Further studies are needed to analyse how the additional quantification of lipoprotein subclasses can improve CV risk stratification and CV disease prediction.

## 1. Introduction

Cardiovascular (CV) diseases are the leading cause of morbidity and mortality worldwide. Lipids and lipoproteins contribute to the development of atherosclerosis as a major underlying pathology of CV diseases [1]. A broad range of evidence exists that especially high low-density lipoprotein cholesterol (LDL-C) and low high-density lipoprotein cholesterol (HDL-C) levels contribute to the manifestations of atherosclerosis [2]. A reduction in LDL-C per every mmol/L has been associated with 22% relative reduction in major CV [3] and 23% of major vascular events [4]. Data from the Framingham Offspring cohort study demonstrated a dose-dependent association between higher non-HDL-C levels and coronary heart disease risk [5]. Even a moderate elevation of non-HDL-C levels has been associated with higher CV risk, which sparked discussions about whether a more aggressive lipid-lowering therapy in primary prevention would be beneficial [5]. Duncan et al. also showed higher risk for atherosclerotic CV diseases with higher LDL-C levels in 3875 participants from the Framingham Offspring cohort. The same study also reported higher risk of incidence of atherosclerotic CV diseases in participants with very low, low, or stable HDL-C levels, compared to participants with consistently high and increasing HDL-C levels [6]. HDL-C in general is known for its athero- and cardioprotective properties. Several years ago, the Framingham Study reported associations of higher HDL-C levels and lower incidence of coronary artery disease [7]. Soppert et al. recently summarized potential mechanisms of how HDL-C protects the vascular system [2]. However, HDL-C elevation with pharmacological therapy failed to reduce risk for major adverse CV events in several studies [8,9,10], which shows the complexity of lipids and lipoprotein particles and their role in CV diseases.

Early detection of vascular dysfunction, as a first step for the development of CV diseases, is considered a key target in CV disease prevention. Several micro- and macrovascular biomarkers exist to non-invasively investigate vascular health or dysfunction. Arteriolar (CRAE) and venular retinal vessel diameters (CRVE) have been shown to independently predict CV outcomes [11,12,13] as well as CV mortality [14]. CV prevention strategies, such as an active lifestyle, have been shown to improve microvascular health, quantified by retinal vessel imaging [15,16]. We have recently published normative data and standard operating procedures to standardize the quantification of retinal vessel diameters as a biomarker to detect microvascular health [17]. Quantification of central pulse wave velocity (PWV) is known as the “gold-standard” method to investigate arterial stiffness in the macrovascular system. PWV is an independent predictor of CV morbidity and mortality in the general population [18,19], elderly subjects [20] and CV risk patients [21]. A meta-analysis of 11 longitudinal studies and 5648 subjects reported that an increase in central PWV by one m/s was associated with an increase in CV and all-cause mortality of 15% [22].

We have recently investigated the association of HDL-C, LDL-C, and the total metabolic profile with micro- and macrovascular health in the EXAMIN AGE cohort [23]. We found that specific metabolites appeared to link traditional CV risk factors with vascular end organ damage. Interestingly, we found no association between total lipoprotein-bound lipids and micro- as well as macrovascular biomarkers. Additionally, triglycerides, HDL-C and LDL-C explained only up to 3% of the total lipoprotein-bound lipid variation [23]. The aim of this study was to quantify lipoprotein subclasses, as a much more distinct analysis of lipids and lipoprotein particles, and investigate their associations with macro- and microvascular health.

## 2. Results

A total of 158 blood samples from the EXAMIN AGE study were analysed to investigate associations of lipoprotein subclasses and macro- as well as microvascular health. Eighty-five female and seventy-three male participants were included in this study. Sample characteristics are presented in Table 1. An overview of the sample characteristics of 84 patients with at least two cardiovascular risk factors can be found in Supplementary Table S1.

Nuclear Magnetic Resonance (NMR) spectroscopy enables the measurement of the main lipoprotein classes (VLDL, IDL, LDL, and HDL) and their respective subclasses. These can be discriminated by size, density, and lipid composition. The highest number indicates the densest and smallest particle with the lowest lipid content of the respective subclass (B.I.-LISA, Bruker IVDr LIpoprotein Subclass Analysis from Bruker Biospin). A list of all 112 lipoprotein subclasses measured by NMR spectroscopy can be found in Supplementary Table S2. Our results from measuring lipoproteins with NMR spectroscopy are in good accordance with classical methods for lipoprotein measurement of all 158 blood samples (see Table 1). The linear correlations between methods are high for all main lipid parameters (total HDL-C: slope = 0.78, r = 0.95; total LDL-C: slope = 0.96, r = 0.89; total blood triglycerides: slope = 0.96, r = 0.97).

### Associations of Vascular Health and Lipoprotein Subclasses

Nineteen lipoprotein subclasses showed statistically significant associations with microvascular and nine with macrovascular health parameters (Table 2 and Table 3).

HDCH, HDFC and H1FC showed statistically significant associations with micro- and macrovascular biomarkers. All 25 lipoprotein subclasses that showed statistically significant associations with vascular biomarkers together explained up to 26% (R^2^ = 0.26, F(29,121) = 2.80, *p* < 0.001) of variation in micro- and 12% (R^2^ = 0.12, F(29,124) = 1.70, *p* = 0.025) of variation in macrovascular health (Table 4). The results of further linear regression models for macro- and microvascular biomarkers as dependent variables, and the following risk factors as predictors, are shown in Table 4: HDL-C, LDL-C, and triglycerides, and previously defined CV risk factors consisting of smoking status, diabetes, hypertension, and body mass index (BMI) as well as physical fitness, corrected for age and sex.

## 3. Discussion

Lipoprotein subclasses showed associations with micro- and macrovascular biomarkers and explained up to 26% of variation in micro- and 12% of variation in macrovascular health in our cohort. HDL-C, LDL-C, and triglycerides together explained only 13% of micro- and 8% of macrovascular variations.

HDL-C and LDL-C are established circulating CV biomarkers that contribute to the development and manifestation of atherosclerosis as previously described [2]. Interestingly, the 25 lipoprotein subclasses showing statistically significant associations with micro- or macrovascular biomarkers together explained variations in vascular health more precisely compared to the common combined measurements of HDL-C, LDL-C, and triglycerides. The lack of sensitivity to predict CV events by analysing HDL-C and LDL-C has previously been shown. Albers et al. demonstrated that HDL subclasses were associated with CV events in the AIM-HIGH clinical trial, whereas HDL-C showed no associations [24]. Chaudhary et al. also showed that HDL-C subclasses improved the prediction of coronary artery disease in patients on statin therapy compared to a traditional lipid panel [25]. Results from the Atherosclerosis Risk in Communities (ARIC) study demonstrated that small-density LDL-C predicted the incidence of coronary heart diseases independent of age, ethnicity, and gender. Interestingly, large buoyant LDL-C showed no concentration-dependent association with future coronary heart disease events in this large cohort study with 11,419 participants [26].

### 3.1. Lipoprotein Subclasses and Microvascular Health

Several HDL and LDL subclasses exist that differ by density, function, and relationship to diseases [27]. Subclasses of HDL, LDL, intermediate-density lipoprotein (IDL), and very-low-density lipoprotein (VLDL), as well as the ratio of Apo-B100/Apo-A1 and LDL/HDL, all showed statistically significant associations with microvascular biomarkers in our study. In particular, CRAE narrowing and CRVE widening, resulting in a low AVR, have previously been associated with CV risk factors such as hypertension [28,29,30], diabetes [29], and obesity [31,32] and have been shown to be predictive for CV outcomes [11,12,13]. Higher concentrations of HDL subclasses showed associations with narrower CRVE and higher AVR, both associated with lower CV risk. HDL and its subclasses are known for their atheroprotective functions. Beazer et al. recently summarized these functions, highlighting their anti-inflammatory and vasodilatory properties [33]. In particular, the stimulation of nitric oxide (NO) synthesis, leading to higher NO bioavailability and lower reactive oxidative species, seems to play a key role in mediating microvascular health. We have previously found indirect evidence for the association of exercise-induced improvements of retinal AVR and higher NO bioavailability in obese individuals [34]. Interestingly, triglycerides of HDL, most prominently HDL-3, showed an inverse association with AVR in our current study. A positive association between HDL-3 and CV diseases has previously been demonstrated [35,36,37]. Hemodialysis patients with high HDL-3 levels showed higher incidence of macrovascular events compared to patients with low HDL-3 levels [35]. Patients with acute coronary syndrome showed higher HDL-3 but lower HDL-C levels, indicating that not all HDL-C subclasses are athero- and cardioprotective [36]. Tian et al. also showed that HDL-3 levels were positively associated with plasma triglyceride levels, which supports the assumption that specific HDL subclasses, in our study specifically HDL-3, might be involved in the pathogenesis of vascular disease [36]. These associations might be explained by the high exposure of HDL-3 to triglycerides in general. Subjects with high total triglyceride concentrations also revealed high HDL triglyceride concentrations in our study (r = 0.646). As expected, the ratios of total LDL to HDL particles as well as Apo-B100 (the main protein constituent of LDL and VLDL) to Apo-A1 (the main protein constituent of HDL) were negatively associated with retinal AVR. Since we cannot draw any conclusions on potential cause and consequence in our cross-sectional study, it is also possible that an altered microvascular environment may interfere with a high burden of triglycerides.

### 3.2. Lipoprotein Subclasses and Macrovascular Health

Higher PWV is an independent indicator for stiff arteries and therefore a suitable vascular biomarker to detect macrovascular dysfunction [38]. Several HDL subclasses (total HDL-C, HDL-1, and HDL-2) showed negative associations with PWV, supporting the previously discussed protective properties of HDL and its subclasses for atherosclerotic CV diseases [33]. Interestingly, we identified an inverse association of LDL-2 components (apolipoprotein B-100, cholesterol, and phospholipids) with PWV. In general, LDL and its subclasses, especially small dense LDL-C, were associated with higher CV risk and mortality [39,40,41,42]. Pokharel et al. reported a stronger association of low-density LDL subclasses with incidence of coronary heart disease compared to large and buoyant LDL subclasses [39]. The Québec Cardiovascular Study reported that especially small dense LDL particles predicted the risk of ischemic heart disease in men [40]. El Harchaoui et al. reported an inverse correlation of LDL size with coronary artery disease [41]. Acute ischemic stroke patients showed higher small dense LDL subclasses, such as LDL-3, but lower levels of large-size LDL subclasses, such as LDL-1 or LDL-2, compared to healthy controls [42]. A prospective cohort study with more than 26.000 healthy women and a follow-up period of 13 years reported a positive association of small dense LDL particles, but a negative association of large-size LDL subclasses, with incidence of diabetes [43]. It seems that a shift in LDL subclasses from large-size to small dense subclasses is associated with higher CV risk. Our study showed that large-size LDL (LDL-2) was negatively associated with PWV, but without observing the associations of PWV with small dense LDL-C subclasses. Instead, we found that microvascular health (AVR) was negatively associated with LDL-5, also known as small dense LDL-C. These results highlight the importance of analysing LDL subclasses instead of LDL-C alone to potentially improve CV risk stratification. However, the underlying mechanisms of the negative associations between LDL-2 and LDL-3 and PWV remain unclear. Even though small dense LDL subclasses have previously been characterized with higher oxidative susceptibility or higher binding affinity to the arterial wall compared to large-size LDL subclasses [44], more research is needed to further decode different mechanisms of small- and large-size LDL subclasses in atherosclerosis.

### 3.3. Lipoprotein Subclasses as Suitable Biomarker to Detect Cardiovascular Risk?

Our results support the evidence described above that lipoprotein subclasses have the potential to improve CV risk stratification compared to a standard lipoprotein profile. Lipoprotein subclasses explained considerably more variation in microvascular health than the classic combination of HDL-C, LDL-C, and triglycerides. However, it is still essential to additionally investigate other CV risk factors such as smoking status, diabetes, hypertension, or body mass index. These risk factors explained up to 38% of micro- and 14% of macrovascular variations in our study. In addition, cardiorespiratory fitness (CRF), assessed as peak oxygen uptake, is a strong predictor of vascular alterations. CRF explained 27% of micro- and 36% of macrovascular alterations. Previous studies have summarized the predictive value of CRF for CV disease development [45,46]. Higher levels of exercise and CRF have been associated with better CV risk profiles [47].

### 3.4. Quantifying HDL and LDL Subclasses in Clinical Practice

A recent review compared a range of methods for measurement of LDL subclasses, including ultracentrifugation, gradient gel electrophoresis, high-performance liquid chromatography, and NMR [48]. In conclusion, quantification of small dense LDL by using either of these methods appeared to be limited. NMR spectroscopy, as arguably the most robust method, may provide a valid and practicable means to determine over 100 parameters with a relatively small amount of sample (300 µL). Future methodological developments intending to include lipoprotein subclass determination, as well as measurements of metabolites, may offer affordable and easy-to-use diagnostic approaches that may foster use in daily clinical routine.

### 3.5. Limitations

Our study is a cross-sectional study without the ability to investigate the causality of whether altered lipoprotein blood levels are a cause or a consequence of altered vascular parameters. This will have to be addressed in future studies. Although our study included samples from patients with and without cardiovascular risk, we refrained from further subgroup analyses of patients with and without cardiovascular risk, as the high number of lipid subclasses in the relatively small groups would not allow for a meaningful analysis. It is well known that lipoprotein subclasses are differently involved in various disease states. Our study sample was designed to characterize individuals with and without CV risk factors, with low or high physical fitness levels, reflecting the general population of healthy individuals as well as CV risk patients. However, the results may be sample-specific and may not be transferable to other age- and disease-specific populations.

## 4. Materials and Methods

The EXAMIN AGE study [49] was conducted between 2015 and 2019 in Basel, Switzerland at the Department for Sports, Exercise and Health. The EXAMIN AGE study was realized to investigate the associations of cardiorespiratory fitness and CV disease with macro- and microvascular health in healthy individuals and CV risk patients. In- and exclusion criteria, as well as general study procedures, were described in detail in our published study protocol [49]. Blood serum samples from this study, which were stored at −80 degrees, were shipped with dry ice to the Gottfried Schatz Research Center for Cell Signaling at the Medical University of Graz in 2021. The following procedure was conducted to quantify lipoprotein subclasses in all 158 participants from the cross-sectional part of the EXAMIN AGE study.

### 4.1. Sample Preparation and Lipoprotein Quantification by NMR Spectroscopy

Blood serum lipoproteins were analysed on a Bruker 600 MHz Avance Neo NMR spectrometer using the Bruker IVDr lipoprotein subclass analysis protocol. Samples were processed as described previously [50]. Shortly afterwards, serum samples were thawed on ice, and 330 µL of each sample was mixed with 330 µL of Bruker’s NMR buffer plasma, amongst others containing 3-(trimethylsilyl) propionic acid-2,2,3,3-d4 sodium salt (TSP), which serves as internal 0.0 ppm standard (Bruker, Rheinstetten, Germany). The samples were mixed gently, and 600 µL thereof was transferred into 5 mm SampleJet rack tubes (Bruker). Proton spectra were obtained at a constant temperature of 310 K using a standard Nuclear Overhauser Enhancement Spectroscopy (NOESY) pulse sequence (Bruker: noesygppr1d), a Carr–Purcell–Meiboom–Gill (CPMG) pulse sequence [51,52] with presaturation during the relaxation delay to achieve water suppression (Bruker: cpmgpr1d), and a standard 2D J-resolved (JRES) pulse sequence (Bruker: jresgpprqf) [53,54,55]. Data analysis was carried out using the Bruker IVDr Lipoprotein Subclass Analysis (B.I.LISA™) method. The unequal distribution of lipids between the core and the surface of lipoproteins results in varying anisotropy within the particles, and hence varying chemical shifts of characteristic methyl and methylene protons of both cholesterol(-esters) and fatty acids in the magnetic field of the NMR spectrometer [56]. This allows the identification of lipoprotein subclasses. The superstition of a sum of Lorentzian-shaped curves [57] over the spectrum of each sample then enables their quantification [58,59,60,61,62]. The subclasses are identified by the chemical shifts of typical methyl and methylene groups of their lipids, and the amount of each (sub)class is calculated from the signal amplitude of the terminal methyl groups [63].

### 4.2. Micro- and Macrovascular Health

Procedures to quantify micro- and macrovascular health were previously described in detail in our published study protocol [49]. Briefly, three images from one eye of every participant were taken to quantify retinal microvascular diameters. CRAE, CRVE, and AVR were analysed as previously described [17,49]. The average of CRAE, CRVE, and AVR from three images of every participant was used to quantify microvascular health. Carotid to femoral PWV was quantified after 10 min of rest in a supine position as described previously [64]. The mean value of two valid measurements with a mean difference of ≤1 m/s was used for further calculations.

### 4.3. Statistical Analysis

Sample characteristics were descriptively described by using mean and standard deviation. Associations of lipoprotein subclasses and micro- (CRAE, CRVE, and AVR) and macrovascular parameters (PWV) were calculated with separated linear regression models for every vascular parameter. To control the false-discovery rate, the Benjamini–Hochberg procedure [65] was applied to correct the *p*-values for multiple testing across all 112 models for each vascular biomarker separately. The corrected *p*-values are displayed in the results section. In addition, Pearson’s product-moment correlations were calculated to report the correlation coefficient (r). Four additional linear regression models were calculated with micro- and macrovascular biomarkers as dependent variables, and lipid subclasses (only statistically significant subclasses from previously calculated associations), HDL, LDL, and triglycerides, classic CV risk factors, and CRF (corrected for age and sex) as independent variables, respectively. Classic CV risk factors were defined a priori as smoking status (smoking or non-smoking), diabetes (antidiabetic medication or fasting glucose levels ≥5.6 mmol/L), hypertension (antihypertensive medication or ≥140 mmHg systolic or ≥90 mmHg diastolic blood pressure during 24 h monitoring), and BMI. Residual and Q-Q plots were used to test homoscedasticity and normal distribution. We found no relevant deviations from model assumptions. All statistical tests were performed two-sided with a significance level of <0.05. Statistics were performed and graphs were designed using R version 3.6.1 or later (R Foundation for Statistical Computing, Vienna, Austria).

## 5. Conclusions

HDL, LDL, and triglycerides are established CV risk factors known to contribute to the development and manifestation of systemic atherosclerosis. Our results show that lipoprotein subclasses seem to reflect vascular end organ damage, quantified by retinal microvascular diameters and large artery stiffness. Future research will have to investigate whether quantification of lipoprotein subclasses can improve CV risk stratification and CV risk prediction with the potential to affect clinical decision making.

## Figures and Tables

**Table 1:** Sample characteristics.

Patients’ Characteristics	Mean ± SD
Age (years)	59 ± 7
Height (cm)	169 ± 8
Body mass (kg)	81.8 ± 18.1
Body mass index (kg/m^2^)	28.6 ± 6.0
Waist circumference (cm)	99 ± 16
Fat mass (%)	28.5 ± 13.2
Muscle mass (kg)	29.6 ± 6.4
24 h systolic BP (mmHg)	125 ± 10
24 h diastolic BP (mmHg)	79 ± 7
Fasting glucose (mmol/L)	5.3 ± 1.5
Triglycerides (mmol/L)	1.4 ± 0.9
HDL-C (mmol/L)	1.6 ± 0.5
LDL-C (mmol/L)	3.1 ± 0.8
Hs-CRP (mg/L)	2.6 ± 3.5
PROCAM score	36 ± 10
PROCAM 10 years risk (%)	6.5 ± 6.4
${\dot{V}}_{O2\mathrm{peak}}$ (mL/min/kg)	31 ± 9
CRAE (μm)	174 ± 14
CRVE (μm)	213 ± 17
AVR	0.82 ± 0.06
PWV (m/s)	7.8 ± 1.7

Abbreviations: BP, blood pressure; HDL-C, high-density lipoprotein cholesterol; LDL-C, low-density lipoprotein cholesterol; Hs-CRP, high-sensitive C-reactive protein; ${\dot{V}}_{O2\mathrm{peak}}$, peak oxygen uptake; CRAE, central retinal arteriolar equivalent; CRVE, central retinal venular equivalent; AVR, arteriolar-to-venular diameter ratio; PWV, pulse wave velocity; SD, standard deviation.

**Table 2:** Associations of lipoprotein subclasses and microvascular health.

Parameter	Microvascular Parameter	R	Corrected *p*-Value	Molecular Class	Lipoprotein (Sub)Classes
**HDL**					
HDCH	AVR	0.324	0.006	Cholesterol	total HDL
HDFC	AVR	0.379	<0.001	Free cholesterol	total HDL
HDFC	CRVE	−0.305	0.016	Free cholesterol	total HDL
H1A1	AVR	0.385	<0.001	Apolipoprotein-A1	HDL-1
H1A1	CRVE	−0.306	0.015	Apolipoprotein-A1	HDL-1
H1A2	AVR	0.341	0.002	Apolipoprotein-A2	HDL-1
H1A2	CRVE	−0.295	0.026	Apolipoprotein-A2	HDL-1
H1CH	AVR	0.383	<0.001	Cholesterol	HDL-1
H1FC	AVR	0.404	<0.001	Free cholesterol	HDL-1
H1FC	CRVE	−0.323	0.006	Free cholesterol	HDL-1
H1PL	AVR	0.376	<0.001	Phospholipids	HDL-1
H1PL	CRVE	−0.287	0.040	Phospholipids	HDL-1
H3TG	AVR	−0.286	0.041	Triglycerides	HDL-3
**LDL**					
LDTG	AVR	−0.304	0.017	Triglycerides	total LDL
L4TG	AVR	−0.301	0.020	Triglycerides	LDL-4
L5AB	AVR	−0.291	0.032	Apoliprotein B-100	LDL-5
L5TG	AVR	−0.299	0.021	Triglycerides	LDL-5
**IDL**					
IDAB	AVR	−0.313	0.010	Apoliprotein B-100	IDL
IDFC	AVR	−0.287	0.040	Free cholesterol	IDL
**VLDL**					
VLAB	AVR	-0.285	0.044	Apoliprotein B-100	total VLDL
V1FC	AVR	−0.284	0.047	Free cholesterol	VLDL-1
V1PL	AVR	−0.287	0.040	Phospholipids	VLDL-1
**TP**					
ABA1	AVR	−0.337	0.003	Apo-B100/Apo-A1	Particle number ratio
LDHD	AVR	−0.290	0.034	LDL/HDL	Particle number ratio

Abbreviations: R, correlation coefficient; HDL, high-density lipoprotein; LDL, low-density lipoprotein; IDL, intermediate-density lipoprotein; VLDL, very-low-density lipoprotein cholesterol; TP, total particles; AVR, arterio-to-venular diameter ratio; CRVE, central retinal venular diameter equivalent.

**Table 3:** Associations of lipoprotein subclasses and macrovascular health.

Parameter	Macrovascular Parameter	R	Corrected *p*-Value	Molecular Class	Lipoprotein (Sub)Classes
HDL					
HDCH	PWV	−0.281	0.046	Cholesterol	total HDL
HDFC	PWV	−0.288	0.032	Free cholesterol	total HDL
H1FC	PWV	−0.306	0.013	Free cholesterol	HDL-1
H2FC	PWV	−0.297	0.021	Free cholesterol	HDL-2
LDL					
L2AB	PWV	−0.286	0.035	Apoliprotein B-100	LDL-2
L2CH	PWV	−0.292	0.027	Cholesterol	LDL-2
L2FC	PWV	−0.309	0.011	Free Cholesterol	LDL-2
L2PL	PWV	−0.299	0.018	Phospholipids	LDL-2
L3FC	PWV	−0.302	0.016	Free Cholesterol	LDL-3

Abbreviations: R, correlation coefficient; HDL, high-density lipoprotein cholesterol; LDL, low-density lipoprotein; PWV, pulse wave velocity.

**Table 4:** Linear regression models.

Dependent Variable	Predictor	F-Statistic (df1, df2, F-Value)	*p*-Value	Adjusted R^2^
AVR	25 lipoprotein subclasses *	29,121 = 2.80	<0.001	0.26
	HDL-C, LDL-C and triglycerides	3143 = 8.42	<0.001	0.13
	classic CV risk factors ^¥^	4146 = 23.6	<0.001	0.38
	${\dot{V}}_{O2\mathrm{peak}}$ ^£^	3147 = 19.36	<0.001	0.27
CRVE	25 lipoprotein subclasses *	29,121 = 2.08	0.003	0.17
	HDL-C, LDL-C and triglycerides	3143 = 3.44	0.048	0.05
	classic CV risk factors ^¥^	4146 = 5.68	<0.001	0.11
	${\dot{V}}_{O2\mathrm{peak}}$ ^£^	3147 = 6.43	<0.001	0.10
CRAE	25 lipoprotein subclasses *	29,121 = 1.08	0.375	0.02
	HDL-C, LDL-C and triglycerides	3143 = 0.65	0.582	0.01
	classic CV risk factors ^¥^	4146 = 1.43	0.227	0.01
	${\dot{V}}_{O2\mathrm{peak}}$ ^£^	3147 = 0.05	0.421	0.00
PWV	25 lipoprotein subclasses *	29,124 = 1.70	0.025	0.12
	HDL-C, LDL-C and triglycerides	3145 = 5.46	0.001	0.08
	classic CV risk factors ^¥^	4149 = 7.46	<0.001	0.14
	${\dot{V}}_{O2\mathrm{peak}}$ ^£^	3150 = 29.28	<0.001	0.36

Abbreviations: AVR, arterio-to-venular diameter ratio; CRVE, central retinal venular diameter equivalent; CRAE, central retinal arteriolar diameter equivalent; PWV, pulse wave velocity; HDL-C, high-density lipoprotein cholesterol; LDL-C, low-density lipoprotein cholesterol; CV, cardiovascular; ${\dot{V}}_{O2\mathrm{peak}}$, peak oxygen uptake; * 25 lipoprotein subclasses that showed statistically significant associations with vascular biomarkers; ^¥^ previously defined CV risk factors consisting of smoking status, diabetes, hypertension, and body-mass index; ^£^ corrected for age and sex.

## Data Availability

The data presented in this study are available on request from the corresponding author.

## Supplementary Materials

The following supporting information can be downloaded at: https://www.mdpi.com/article/10.3390/molecules27154760/s1, Table S1: Sample characteristics of 84 patients with at least two cardiovascular risk factors. Table S2: A list of all 112 parameters measured by NMR spectroscopy.

## Sample Availability

Serum samples are not available from the authors.
