# Supplementary material for: Lipoprotein Subclasses Independently Contribute to Subclinical Variance of Microvascular and Macrovascular Health
Source: Molecules. 2022 Jul 25;27(15):4760. doi: 10.3390/molecules27154760 (PMC9332701; doi:10.3390/molecules27154760)
Supplement: Supplementary file 1 [file molecules-27-04760-s001.zip › molecules-1813018-supplementary.pdf]

molecules

Supplementary Materials

Lipoprotein Subclasses Independently Contribute to Subclinical Variance of Microvascular and Macrovascular Health

Lukas Streese [1,†], Hansjörg Habisch [2,†], Arne Deiseroth [1], Justin Carrard [1], Denis Infanger [1], Arno Schmidt-Trucksäss [1], Tobias Madl [2,3,*,‡] and Henner Hanssen [1,‡]

1 Department of Sport, Exercise and Health, Medical Faculty, University of Basel, 4052 Basel, Switzerland; lukas.streese@unibas.ch (L.S.); arne.deiseroth@unibas.ch (A.D.); justin.carrard@unibas.ch (J.C.); denis.infanger@unibas.ch (D.I.); arno.schmidt-trucksaess@unibas.ch (A.S.-T.); henner.hanssen@unibas.ch (H.H.)
2 Gottfried Schatz Research Center for Cell Signaling, Metabolism and Aging, Molecular Biology and Biochemistry, Medical University of Graz, 8010 Graz, Austria; hansjoerg.habisch@medunigraz.at
3 BioTechMed Graz, 8010 Graz, Austria
* Correspondence: tobias.madl@medunigraz.at; Tel.: +43-316-385-71972
† These authors contributed equally to this work as shared first authorship.
‡ These authors contributed equally to this work as shared senior authorship.

Table S1. Sample characteristics of 84 patients with at least two cardiovascular risk factors.

Patients' characteristics	Risk Cohort Mean±SD
Sex (f/m)	42/42
Age (years)	59±6
Height (cm)	169±8
Body mass (kg)	94.7±14.0
Body mass index (kg/m^2)	33.2±4.1
Waist circumference (cm)	112±11
Fat mass (%)	40.1±8.2
Muscle mass (kg)	31.6±6.9
24h systolic BP (mmHg)	130±11
24h diastolic BP (mmHg)	81±8
Fasting glucose (mmol/l)	5.8±1.8
Triglycerides (mmol/l)	1.8±1.1
HDL-C (mmol/l)	1.3±0.3
LDL-C (mmol/l)	3.2±0.8
Hs-CRP (mg/l)	3.6±4.1
PROCAM score	42±10
PROCAM 10 years risk (%)	9.5±7.3
VO2peak (ml/min/kg)	26±4
CRAE (µm)	171±14
CRVE (µm)	218±16
AVR	0.79±0.05
PWV (m/s)	8.3±1.5

Abbreviations: BP, blood pressure; HDL-C, high-density lipoprotein cholesterol; LDL-C, low-density lipoprotein cholesterol; Hs-CRP, high-sensitive C-reactive protein; VO2peak, peak oxygen uptake; CRAE, central retinal arteriolar equivalent; CRVE, central retinal venular equivalent; AVR, arteriolar-to-venular diameter ratio; PWV, pulse wave velocity; SD, standard deviation.

Table S2. A list of all 112 lipoprotein-related parameters measured by NMR spectroscopy.

**All analytes and 95% confidence intervals for the model of the IVDr Lipoprotein Subclass Analysis (B.I.LISA™) method of Bruker.**						**Quantiles measured in this study cohort**		
**analyte**	**unit**	**name**	**range min**	**-**	**range max**	**2.5%**	**50%**	**97.5%**
TPTG	mg/dL	total triglycerides	53	-	490	53	113	460
TPCH	mg/dL	total cholesterol	140	-	341	151	233	327
LDCH	mg/dL	LDL cholesterol	55	-	227	72	135	202
HDCH	mg/dL	HDL cholesterol	35	-	96	36	61	96
TPA1	mg/dL	total Apo-A1	112	-	217	117	165	219
TPA2	mg/dL	total Apo-A2	24	-	48	29	38	51
TPAB	mg/dL	total Apo-B100	48	-	160	64	105	155
LDHD	-/-	LDL-cholesterol/HDL-cholesterol ratio	0.98	-	4.08	1.0	2.2	3.9
ABA1	-/-	Apo-B100/Apo-A1 ratio	0.3	-	1.07	0.4	0.6	1.1
TBPN	nmol/L	total particle number	876	-	2908	1170	1902	2812
VLPN	nmol/L	VLDL particle number	50	-	473	46	153	490
IDPN	nmol/L	IDL particle number	36	-	316	36	99	221
LDPN	nmol/L	LDL particle number	760	-	2560	926	1602	2272
L1PN	nmol/L	LDL-1 particle number	98	-	567	89	210	375
L2PN	nmol/L	LDL-2 particle number	47	-	427	21	148	285
L3PN	nmol/L	LDL-3 particle number	51	-	499	22	188	334
L4PN	nmol/L	LDL-4 particle number	77	-	577	48	249	419
L5PN	nmol/L	LDL-5 particle number	86	-	615	158	345	584
L6PN	nmol/L	LDL-6 particle number	91	-	815	308	461	972
VLTG	mg/dL	VLDL triglycerides	21	-	336	34	85	330
IDTG	mg/dL	IDL triglycerides	5	-	100	1.6	10	68
LDTG	mg/dL	LDL triglycerides	12	-	45	12.3	20	41
HDTG	mg/dL	HDL triglycerides	7	-	29	6.7	12	20
VLCH	mg/dL	VLDL cholesterol	5	-	77	3.9	19	67
IDCH	mg/dL	IDL cholesterol	4	-	50	3.1	14	39
VLFC	mg/dL	VLDL free cholesterol	3	-	33	3.0	10	32
IDFC	mg/dL	IDL free cholesterol	1	-	14	0.9	4.0	11
LDFC	mg/dL	LDL free cholesterol	17	-	63	21	39	56
HDFC	mg/dL	HDL free cholesterol	7	-	27	6.2	14	23
VLPL	mg/dL	VLDL phospholipids	6	-	68	6.9	22	72
IDPL	mg/dL	IDL phospholipids	3	-	33	3.3	8.2	26
LDPL	mg/dL	LDL phospholipids	37	-	121	45	73	105
HDPL	mg/dL	HDL phospholipids	57	-	136	52	81	123
HDA1	mg/dL	HDL Apo-A1	110	-	222	115	164	223
HDA2	mg/dL	HDL Apo-A2	25	-	48	31	39	51
VLAB	mg/dL	VLDL Apo-B100	3	-	26	2.6	8.4	27
IDAB	mg/dL	IDL Apo-B100	2	-	17	2.0	5.5	12
LDAB	mg/dL	LDL Apo-B100	42	-	141	51	88	125
V1TG	mg/dL	VLDL-1 triglycerides	6	-	212	16	45	177
V2TG	mg/dL	VLDL-2 triglycerides	3	-	67	1.5	11	60
V3TG	mg/dL	VLDL-3 triglycerides	2	-	49	1.2	10	47
V4TG	mg/dL	VLDL-4 triglycerides	3	-	28	2.7	9.3	24
V5TG	mg/dL	VLDL-5 triglycerides	1	-	7	1.5	2.9	4.9
V1CH	mg/dL	VLDL-1 cholsesterol	1	-	35	1.3	5.8	29
V2CH	mg/dL	VLDL-2 cholsesterol	0	-	15	0.2	2.3	13
**analyte**	**unit**	**name**	**range min**	**-**	**range max**	**2.5%**	**50%**	**97.5%**

V3CH	mg/dL	VLDL-3 cholsesterol	0	-	16	0.4	3.5	14
V4CH	mg/dL	VLDL-4 cholsesterol	1	-	15	1.5	5.7	15
V5CH	mg/dL	VLDL-5 cholsesterol	0	-	4	0.2	1.5	3.0
V1FC	mg/dL	VLDL-1 free cholsesterol	0	-	13	0.0	2.3	12
V2FC	mg/dL	VLDL-2 free cholsesterol	0	-	7	0.0	0.8	6.2
V3FC	mg/dL	VLDL-3 free cholsesterol	0	-	8	0.1	1.4	7.3
V4FC	mg/dL	VLDL-4 free cholsesterol	0	-	7	0.4	2.3	6.0
V5FC	mg/dL	VLDL-5 free cholsesterol	0	-	2	0.0	0.4	1.5
V1PL	mg/dL	VLDL-1 phospholipids	1	-	32	2.0	6.8	28
V2PL	mg/dL	VLDL-2 phospholipids	1	-	15	0.5	2.9	13
V3PL	mg/dL	VLDL-3 phospholipids	1	-	14	0.5	3.6	14
V4PL	mg/dL	VLDL-4 phospholipids	2	-	13	1.6	4.9	11
V5PL	mg/dL	VLDL-5 phospholipids	0	-	5	0.4	1.8	3.5
L1TG	mg/dL	LDL-1 triglycerides	3	-	14	1.3	4.5	13
L2TG	mg/dL	LDL-2 triglycerides	1	-	6	0.4	1.6	3.4
L3TG	mg/dL	LDL-3 triglycerides	1	-	6	1.2	2.6	4.4
L4TG	mg/dL	LDL-4 triglycerides	1	-	8	0.8	2.7	5.5
L5TG	mg/dL	LDL-5 triglycerides	1	-	9	1.3	3.3	7.0
L6TG	mg/dL	LDL-2 triglycerides	1	-	13	2.4	4.5	11
L1CH	mg/dL	LDL-1 cholsesterol	8	-	59	7.6	22	41
L2CH	mg/dL	LDL-2 cholsesterol	2	-	48	0.0	14	31
L3CH	mg/dL	LDL-3 cholsesterol	3	-	46	0.0	18	32
L4CH	mg/dL	LDL-4 cholsesterol	4	-	49	0.7	22	37
L5CH	mg/dL	LDL-5 cholsesterol	5	-	49	12	27	47
L6CH	mg/dL	LDL-6 cholsesterol	6	-	54	21	32	59
L1FC	mg/dL	LDL-1 free cholsesterol	2	-	17	2.8	6.9	12
L2FC	mg/dL	LDL-2 free cholsesterol	1	-	14	0.4	4.7	10
L3FC	mg/dL	LDL-3 free cholsesterol	1	-	13	1.0	5.6	10
L4FC	mg/dL	LDL-4 free cholsesterol	1	-	12	1.6	6.5	10
L5FC	mg/dL	LDL-5 free cholsesterol	2	-	13	4.1	7.3	12
L6FC	mg/dL	LDL-6 free cholsesterol	2	-	12	5.4	8.8	15
L1PL	mg/dL	LDL-1 phospholipids	6	-	30	5.2	12	22
L2PL	mg/dL	LDL-2 phospholipids	2	-	25	0.4	8.0	16
L3PL	mg/dL	LDL-3 phospholipids	2	-	24	1.1	10	17
L4PL	mg/dL	LDL-4 phospholipids	3	-	25	1.7	12	19
L5PL	mg/dL	LDL-5 phospholipids	4	-	25	6.5	14	24
L6PL	mg/dL	LDL-6 phospholipids	4	-	28	12	17	31
L1AB	mg/dL	LDL-1 Apo-B100	5	-	31	4.9	12	21
L2AB	mg/dL	LDL-2 Apo-B100	3	-	23	1.1	8.1	16
L3AB	mg/dL	LDL-3 Apo-B100	3	-	27	1.2	10	18
L4AB	mg/dL	LDL-4 Apo-B100	4	-	32	2.6	14	23
L5AB	mg/dL	LDL-5 Apo-B100	5	-	34	8.7	19	32
L6AB	mg/dL	LDL-6 Apo-B100	5	-	45	17	25	53
H1TG	mg/dL	HDL-1 triglycerides	1	-	12	1.3	3.2	6.8
H2TG	mg/dL	HDL-2 triglycerides	1	-	5	0.9	1.7	2.9
H3TG	mg/dL	HDL-3 triglycerides	1	-	5	1.1	2.2	4.3
H4TG	mg/dL	HDL-4 triglycerides	2	-	8	1.7	4.0	7.5
H1CH	mg/dL	HDL-1 cholesterol	6	-	46	5.2	15	42
H2CH	mg/dL	HDL-2 cholesterol	4	-	16	3.7	8.4	14
H3CH	mg/dL	HDL-3 cholesterol	7	-	19	6.5	11	16
**analyte**	**unit**	**name**	**range min**	**-**	**range max**	**2.5%**	**50%**	**97.5%**

H4CH	mg/dL	HDL-4 cholesterol	11	-	30	18	25	33
H1FC	mg/dL	HDL-1 free cholesterol	1	-	12	1.4	4.3	11
H2FC	mg/dL	HDL-2 free cholesterol	1	-	5	0.9	2.3	3.7
H3FC	mg/dL	HDL-3 free cholesterol	1	-	5	1.4	2.8	4.3
H4FC	mg/dL	HDL-4 free cholesterol	2	-	9	3.3	5.3	7.6
H1PL	mg/dL	HDL-1 phospholipids	8	-	57	6.4	17	50
H2PL	mg/dL	HDL-2 phospholipids	7	-	27	6.1	13	23
H3PL	mg/dL	HDL-3 phospholipids	12	-	32	10	18	26
H4PL	mg/dL	HDL-4 phospholipids	20	-	44	24	32	45
H1A1	mg/dL	HDL-1 Apo-A1	6	-	75	6.2	21	68
H2A1	mg/dL	HDL-2 Apo-A1	10	-	36	12	20	31
H3A1	mg/dL	HDL-3 Apo-A1	18	-	47	18	29	41
H4A1	mg/dL	HDL-4 Apo-A1	56	-	110	68	91	119
H1A2	mg/dL	HDL-1 Apo-A2	1	-	8	0.7	2.4	6.4
H2A2	mg/dL	HDL-2 Apo-A2	2	-	8	1.8	3.7	5.9
H3A2	mg/dL	HDL-3 Apo-A2	5	-	12	4.6	7.1	11
H4A2	mg/dL	HDL-4 Apo-A2	12	-	30	17	25	34
